# Computational Designed and Optimized Liposomal Curcumin-Embedded Bifunctional Cross-Linked Hydrogels for Wound Healing

**DOI:** 10.3390/gels10090598

**Published:** 2024-09-18

**Authors:** Chaiyakarn Pornpitchanarong, Khin Cho Aye, Kwanputtha Arunprasert, Praneet Opanasopit, Prasopchai Patrojanasophon

**Affiliations:** 1 Pharmaceutical Development of Green Innovations Group (PDGIG), Faculty of Pharmacy, Silpakorn University, Nakhon Pathom 73000, Thailand; pornpitchanaron_c@su.ac.th (C.P.); aye_k@su.ac.th (K.C.A.); arunprasert_k@su.ac.th (K.A.); opanasopit_p@su.ac.th (P.O.); 2 Research and Innovation Center for Advanced Therapy Medicinal Products, Faculty of Pharmacy, Silpakorn University, Nakhon Pathom 73000, Thailand; 3 Health Intervention and Technology Assessment Program (HITAP), Ministry of Public Health, Nonthaburi 11000, Thailand

**Keywords:** curcumin, liposomes, hydrogel, wound dressings, hyaluronic acid, poly(vinyl alcohol), optimization

## Abstract

Curcumin (CUR) bifunctional cross-linked nanocomposite hydrogels are presented as an efficient method for CUR delivery in wound healing. CUR-loaded liposomes (CUR-Ls) were optimized using the Box–Behnken design to augment particle size, size distribution, zeta potential, and CUR concentration. The antioxidant activity and cytotoxicity of CUR-Ls were assessed. Hyaluronic acid (HA)/poly(vinyl alcohol) (PVA) hydrogels were optimized with a central composite design; then, poly(N-vinylpyrrolidone-co-itaconic acid) (PNVP-ITA) was synthesized to enrich the properties of the hydrogels. The drug release kinetics of the CUR-L@HA/PVA/PNVP-ITA hydrogels were studied. Skin recovery was investigated in vivo on rat dorsal skin. The optimized CUR-Ls were constructed from 2.7% Tween^®^ 20, 0.04% oleic acid, and 8.1% CUR, yielding nano-CUR-L with a narrow size distribution, negative surface charge, and CUR content of 19.92 ± 0.54 µg/mg. CUR-Ls improved the antioxidant effects of CUR. The optimized hydrogel contained 5% HA and 10% PVA. PNVP-ITA improved the properties of the hydrogels via enhanced cross-linking. CUR-Ls exhibited a more rapid release than CUR, whereas the hydrogels enhanced CUR release via a diffusion-controlled mechanism. CUR-L@HA/PVA/PNVP-ITA hydrogels improved the skin recovery rate compared to the commercial patch after 5 days. Therefore, the optimized CUR-L@HA/PVA/PNVP-ITA hydrogels facilitated skin recovery and could be a promising nanocomposite for wound dressings.

## 1. Introduction

People suffer from wounds due to their daily activities, occupation, or accidents, and the number of cases is increasing worldwide. The prevalence of acute and chronic wounds was 10.55 and 4.48 per 1000 of the population, respectively [1]. The global wound care market is expected to grow at an annual rate of 4.61% between 2023 and 2030 [2]. Severe acute wounds caused by trauma or surgery typically do not self-heal, and chronic wounds such as diabetic ulcers and pressure sores are more frequently found. Wound management remains a major clinical problem [3,4]. Despite the widespread use of available treatment options, such as bandages, gauze, and cotton wool, these materials still rely on passive healing, making them less effective in the management of severe or chronic wounds. Currently, appropriate wound management is a significant clinical concern, and there has been a substantial increase in the demand for wound care. Several innovative and efficient wound dressing materials, including hydrocolloids, nanofibers, nanoparticles, and hydrogels, have been created to increase therapeutic efficacy by keeping the wound moist, absorbing exudates, and preventing infections [5,6,7,8].

Hydrogels are highly hydrated cross-linked polymer networks that maintain the environment of natural soft tissues, making them desirable for wound dressings [9,10]. Moreover, hydrogel dressings can reduce pain at rest, mobility, and tissue injuries upon dressing removal, while reducing wound infection when appropriate materials and compositions are used. Furthermore, the moist environment provided by hydrogels facilitates hydration and enables fast and effective distribution of the added active component [11]. Additionally, it can efficiently absorb exudates through water absorption and hydrogel network swelling [12]. Hydrogels can be fabricated using different methods, including free radical polymerization, physical cross-linking, chemical cross-linking, and three-dimensional printing. [13] Several studies have shown that poly(vinyl alcohol) (PVA) is a promising synthetic polymer used in the fabrication process [14,15,16]. PVA is a biodegradable and water-soluble polymer with inert characteristics. Hydroxyl side chains have good biocompatibility and enable PVA to crosslink through various physical and chemical methods. Notably, the polymer is inexpensive and stable, with tunable mechanical strength [17]. However, the toughness may still be unsuitable for biomedical applications; when the degree of cross-linking is insufficient, adding nanoparticulate systems (nanocomposite hydrogels) or creating a double polymeric network may be required to enhance the mechanical suitability [18,19]. Hyaluronic acid (HA) is a natural polysaccharide and is beneficial as a hydrogel component. Owing to HA being biocompatible, biodegradable, and non-immunogenic, and having the ability to interact with cells and growth factors with high water retention, it has emerged as a beneficial biomaterial for bone and tissue engineering [20,21,22]. Physical cross-linking is a convenient approach for fabricating hydrogels, especially freeze–thaw cycles, in which no special technique, instrument, or reagent is required. In contrast, chemical cross-linking provides a stronger bond, leading to improved mechanical properties and stability. Thus, the metal coordination complex is a safe chemical crosslink procedure suitable for biomedical applications [23,24]. The hydroxyls located in the PVA and HA structures facilitate the formation of metal coordinate covalent bonds, especially when carbonyl- and carboxyl-rich polymers are present [25]. In the present study, poly(*N*-vinylpyrrolidone-co-itaconic acid) (PNVP-ITA) was synthesized for coordination cross-linking to create a compact hydrogel with improved toughness and durability. This would make the hydrogel less prone to breakage upon application.

Curcumin (CUR), a potent active substance found in the rhizome of *Curcuma longa* L., has been the target of considerable and ongoing research owing to its biofunctional attributes, including anti-inflammatory, antioxidant, and radical scavenging effects. These effects are crucial for wound healing [26,27]. CUR accelerates wound regeneration by stimulating the synthesis of growth factors involved in the healing process. Moreover, CUR has been reported to affect the inflammatory, proliferative, and remodeling phases of wound healing. In the inflammatory phase, curcumin reduces the expression of pro-inflammatory cytokines, such as tumor necrosis factor-alpha (TNF-α) and interleukin-1 (IL-1). CUR also inhibits nuclear factor κB (NF-κB) by suppressing the activity of kinases such as AKT, PI3K, and IKK involved in various inflammatory pathways. In the latter phases, CUR plays an important role in inducing cell adhesion and proliferation, cellular invasion, and angiogenesis [28]. Poor water solubility and light intolerance have restricted the application of CUR [29,30]. Nanoparticulate systems have been developed for pharmaceutical uses in recent decades to cope with the undesirable features of active compounds, such as a large surface area and entrapment efficiency [31]. Considering their advantages, hydrogels and CUR nanoformulations are beneficial for wound healing.

Liposome formulations have been shown to be useful for increasing the water solubility, stability, and bioactivity of CUR [32]. CUR-loaded liposomes (CUR-Ls) improve CUR solubility, and thus, bioavailability and bioactivity [33]. CUR nanoformulations added to a novel hydrogel with a high water absorption capacity could improve the therapeutic effects of both CUR and the hydrogel. To date, few formulations of hydrogels containing nano-CUR have been studied.

**Scheme 1:**
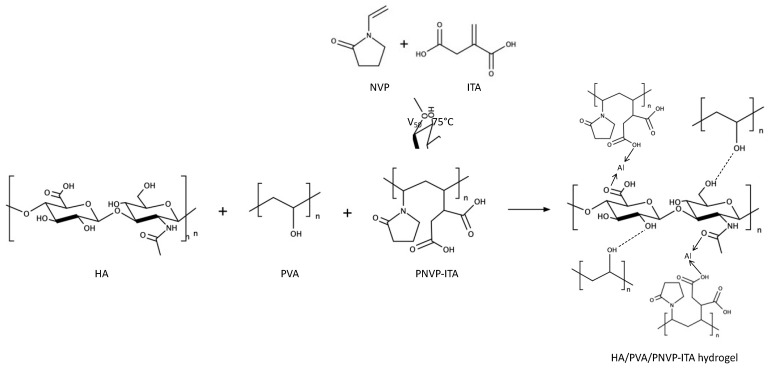
The schematic presentation of the preparation of the HA/PVA/PNVP-ITA bifunctional cross-linked hydrogels through hydrogen bonds (dashed line) and metal coordination complex (arrow).

This study highlights the preparation of CUR-Ls-embedded bifunctional nanocomposite hydrogels. The formulation of CUR-Ls was designed and optimized with computer assistance, aiming to maximize CUR content. Additionally, the HA/PVA hydrogels were optimized through experiments using a central composite design. Then, the optimized CUR-Ls were embedded into the augmented hydrogels strengthened with the newly synthesized PNVP-ITA. Comprehensive examinations were conducted to evaluate the benefits of the designed system in wound healing applications. Moreover, the in vivo wound healing effects of the CUR-L@HA/PVA/PNVP-ITA hydrogels were investigated. HA, PVA, and PNVP-ITA would allow the formation of hydrogel cross-linked through hydrogen bond formation and metal coordinate covalent bond across the hydroxyl-, carbonyl-, and carboxyl-rich polymers. The formulation was developed through a computer-aided approach, creating an optimized CUR nanocomposite hydrogel formulation. Further, the bifunctional cross-linking avenue among the optimized and synthesized polymers, fabricated following Scheme 1, showed the values of multiple interactions to benefit the toughness and suitability of the nanocomposite hydrogel in enriching the wound healing potential of CUR while maintaining its integrity upon application. The computer-aided design and optimization permitted the liposomes and hydrogel formulations to exhibit the desired characteristics, specifically for wound healing applications. This approach could present the efficacy of CUR within hydrogels in wound healing, which has not been widely reported.

## 2. Results and Discussion

### 2.1. Preparation and Optimization of Curcumin-Loaded Liposomes by DoE

CUR-Ls were prepared using the thin-film technique. The Box–Behnken experimental design was used to generate 17 experimental runs from the given liposome composition with various percentages of Tween^®^ 20 (X_1_), oleic acid (X_2_), and CUR content (X_3_). The responses (i.e., particle size (Y_1_), PDI (Y_2_), ZP (Y_3_), and drug content (Y_4_) of the formulated liposomes) were measured (Table 1). The Box–Behnken experimental design is a response surface methodology that allows the prediction of the relationship between multiple variables and their effects on particular outcomes. Especially when three variables are studied, this design is more convenient, yet efficacious, with fewer experimental runs compared to other designs. In addition, the Box–Behnken design avoids combinations where all factors are simultaneously at their maximum or minimum levels, emphasizes midpoints, and allows for better detection of curvature in response surfaces. It was found that the particle sizes and PDI values were in the range of 61–114 nm and 0.01–0.39, respectively. The size of the obtained liposomes was on a considerable nanoscale, and PDI values <0.5 indicated a desirable uniform size [34]. The ZP of the prepared liposomes was negative, measuring between −7 and −45 mV, due to the ionization of oleic acid with a pKa of 9.85 in a pH 7.4 solution [35]. The CUR content (Y4) was in the range of 2–21.8 µg/mg. Apart from the amount of CUR added, Tween^®^ 20 appeared to play a critical role in the structure of liposomes, leading to greater amounts of CUR. As a surfactant, polysorbates improve the drug/lipid ratio, enhance stability, and provide higher encapsulation efficiency [36,37].

ANOVA and multiple regression analyses were conducted to determine the most suitable regression function that describes the relationship between the independent and dependent variables. Table 2 presents the results. All responses showed *p*-values < 0.05, demonstrating a significant relationship between the change in liposome compositions and responses. Data were obtained to fit into different mathematical models, where the particle size (Y_1_) and CUR content (Y_4_) fit suitability to the linear model, and the PDI (Y_2_) and ZP (Y_3_) best fit the quadratic model. The lack of fit *p*-value > 0.05 indicated the adequacy of the multiple regression model, except for the PDI value showing that the functional relationship between the experimental factors and response variable response (Y_2_) could not be described and that the PDI was neglected from the optimization criteria (Table 3). Typically, regression coefficients (R^2^) > 0.6 denotes a reliable model for showing moderate to strong correlation among the variables [38]. The R^2^ values for Y_1_, Y_3_, and Y_4_ were 0.8750, 0.9885, and 0.6240, respectively. These coefficients emphasized the relevance of the predicted models while explaining the relationships. Multiple regression equation models (coded equations) were generated from the interactions of the parameters studied to predict the responses when the input factors varied (Table 2).

**Figure 1:**
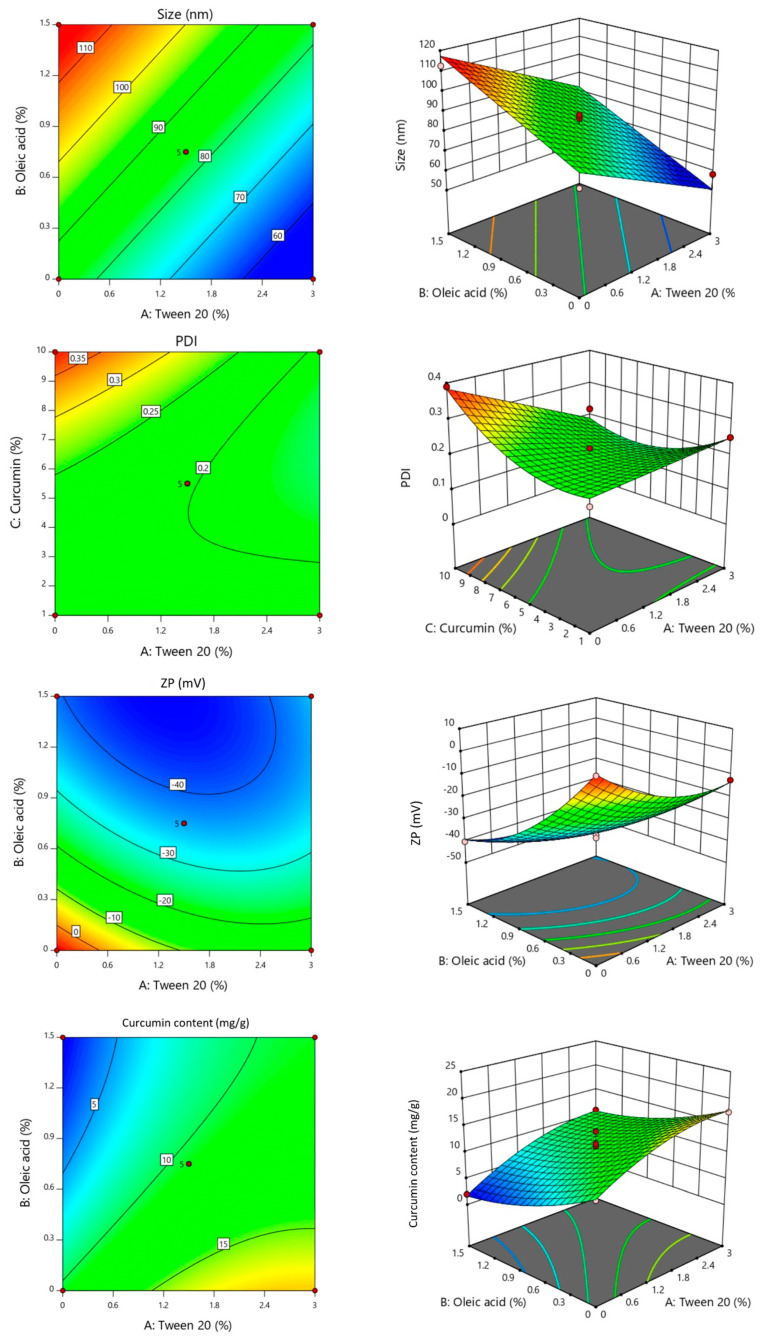
2D and 3D response surface graphs demonstrating the influence of the formulation and process parameters on particle size, PDI, ZP, and CUR content.

The size of liposomes is a crucial parameter impacting drug loading, significantly influencing the pharmacokinetics and pharmacodynamics of drugs [36]. Additionally, it plays a pivotal role in influencing the physicochemical attributes of the drug, including solubility, release kinetics, and permeability [39]. 2D and 3D response surface graphs (Figure 1) were generated to explore parameter interactions and relationships. The regression equation and response surface graphs showed that the quantity of Tween^®^ 20 (X_1_) exhibited a negative impact on particle size (Y_1_); the liposome diameter increased as the amount of Tween^®^ 20 decreased. The reduction in particle size when Tween^®^ 20 was increased was possibly due to the steric repulsion of the particles [36]. Moreover, the addition of a surfactant presents a destabilizing effect within the bilayer. The amphiphilic structure of Tween^®^ 20 enabled an increased interaction between the phospholipid bilayer and the aqueous environment, facilitating the formation of more liposomes with a smaller diameter [40]. Conversely, increasing the amounts of oleic acid (X_2_) and CUR (X_3_) resulted in an increase in particle size due to the incorporation of CUR into the liposome bilayers. The PDI (Y_2_) determines the homogeneity of the size of the liposome formulations. The value was positively affected by all compositions according to the PDI regression equation and response surface graphs. However, the multiple regression equation model showed that the interaction of the two factors (X_1_X_3_) led to negative consequences (Table 2), implying an increase in PDI when the amounts of Tween^®^ 20 and CUR were reduced. However, it should be noted that the lack of fit for this parameter was significant. Regarding ZP (Y_3_), the regression equation and response surface graphs illustrated the positive influence of the amount of CUR and the interaction of the two factors (X_1_X_2_ and X_2_X_3_). This trend is correlated with the charge of the stabilizer. Tween^®^ 20 acts as a nonionic surfactant and oleic acid as an anionic stabilizer [41]. Accordingly, increasing the quantities of Tween^®^ 20 and oleic acid resulted in a more negative charge on the particle surface. This negative ion barrier could hinder particle aggregation, growth, or Ostwald ripening, thereby influencing the physical stability of liposomes. Generally, nanoparticles with high positive or negative ZP values exhibit high stability [42]. Last, increased CUR loading was linked to the added quantities of Tween^®^ 20 and CUR, whereas increased oleic acid was associated with decreased CUR content. This is presumably due to the repulsion force between oleic acid carboxylates and the partial negative moment of CUR.

For the optimization of CUR-Ls, a criterion for each parameter was chosen (Table 3). The desired liposomes should be uniform small vesicles with a negative surface charge and high CUR content. Therefore, the proposed criteria for the optimization of CUR-Ls include minimized particle size and maximized CUR content; the ZP of all runs was in the desirable range of negative values according to the experimental runs. PDI values were omitted from the optimization because the correlation between the independent variables was insignificant. The optimized liposome formulation proposed in the experimental design consisted of 2.7% Tween^®^ 20, 0.04% oleic acid, and 8.1% CUR, achieving a desirability score of 1.00. Desirability is an indicator for estimating the multiresponse optimization value. The acceptable and excellent desirability should be in the range of 0.8–1, indicating the outstanding quality of the predicted formulation [43,44]. The optimized CUR-Ls were confirmed by comparing the actual results with the predicted values from the computer-aided optimization responses. The results were comparable with the predicted solution (*p* > 0.05), confirming that the obtained liposome formulation was optimized and could reproduce consistent liposomes to be further embedded into the hydrogels. Accordingly, computationally designed and optimized CUR-Ls were beneficial for establishing a reproducible formulation with predictable and reliable results.

### 2.2. Differential Scanning Calorimetry

**Figure 2:**
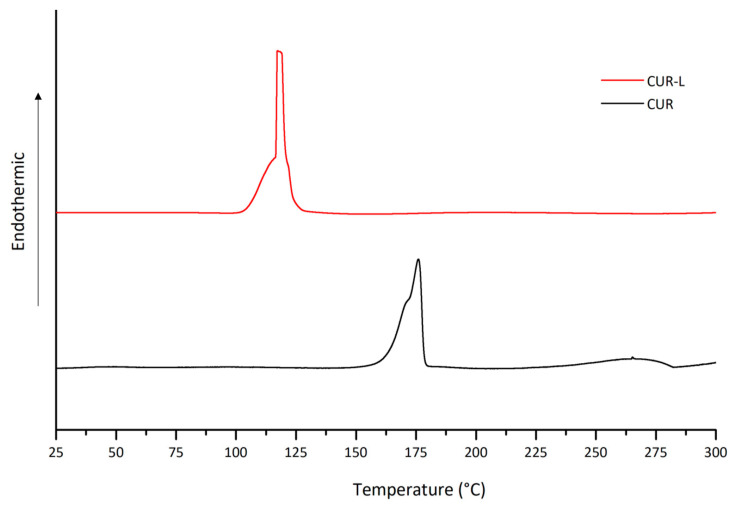
DSC thermogram of CUR and CUR-Ls.

The thermal properties of the CUR-Ls were analyzed using DSC, and the thermogram is presented in Figure 2. The endothermic peak of Cur was present at 175 °C, which suggested the characteristic pattern of crystalline Cur. The peak was found to be absent from the thermal behavior of CUR-Ls, which possibly indicated that the crystallinity of Cur-NSs appeared to shift from the crystalline form of Cur to an amorphous state. The dominant peak at 116 °C of the Cur-L thermogram was due to the presence of Tween^®^ 20, which was similar to that reported in the literature [45]. Several studies have suggested that encapsulating a drug within a liposomal bilayer can lead to the transformation from a crystalline form to an amorphous state. This is because the confined environment within the liposomal bilayer disrupts the regular crystal lattice structure of the drug, preventing it from maintaining its ordered crystalline form [32,46,47]. The amorphous state is particularly beneficial for hydrophobic drugs like Cur, as it improves drug dissolution and enhances efficacy in aqueous environments.

### 2.3. Antioxidant Activity

**Figure 3:**
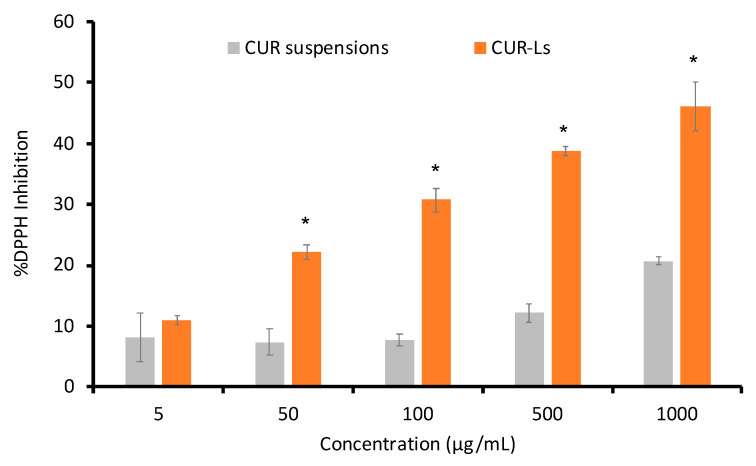
Antioxidant activity of CUR suspensions and CUR-Ls (* Significant difference, *p* < 0.05).

The amount of CUR in the CUR-L formulation directly influenced its antioxidant potential, which was measured using the DPPH assay. Analysis revealed that CUR-Ls at 36.9 µg/mL exhibited a 20% inhibition of DPPH radicals (Figure 3). Conversely, CUR aqueous suspensions required a higher concentration of 970.4 µg/mL to achieve an equivalent DPPH inhibition, whereas ascorbic acid (employed as a positive control) at 1.8 µg/mL showed an inhibition > 20%. Moreover, the antioxidant activity of CUR-Ls was 26.3-fold more potent than that of CUR in an aqueous medium. This is because liposomal curcuminoids are more stable and can be solubilized in aqueous media [48,49]. Hence, the increased solubility of CUR in CUR-Ls greatly influences its antioxidant activity. CUR’s antioxidant potential plays a significant role in wound healing, primarily by reducing inflammation. The effect of CUR on oxidative stress markers is attributed to its specific qualities that target the elimination of reactive oxygen and nitrogen species, metal chelation abilities, and its regulation of various enzymes [50,51]. Its antioxidant efficacy is linked to its high CUR content. The anti-inflammatory effects of CUR have been reported to result from different mechanisms associated with its capacity to scavenge free radicals generated by lipoxygenases [50,51]. Additionally, the molecular structure of CUR was characterized by phenolic groups positioned in the *ortho*- and/or *para*-positions of the aromatic rings, presenting strong electron-shifting properties, which in turn enabled reactive oxygen species scavenging. CUR plays a role in various stages of the healing process, during which its antioxidant effect can specifically improve wound healing [52]. However, the low water solubility of CUR limits its bioavailability and represents a major obstacle to wound healing [53]. Therefore, CUR-Ls may significantly contribute to wound healing owing to their robust antioxidant effects augmented by improved solubility [54].

### 2.4. Cytotoxicity Assessment

**Figure 4:**
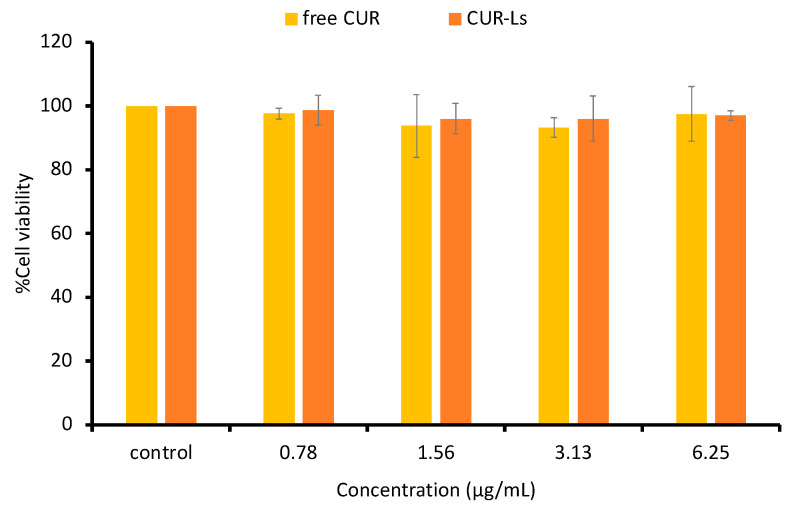
Cytotoxicity evaluation of free CUR and CUR-Ls.

An MTT test was conducted to assess the in vitro cytotoxicity of the liposomal formulation. HSF cells were exposed to sample solutions within serum-free culture medium for 24 h using CUR concentrations of 0.78–6.25 µg/mL. Figure 4 illustrates the cell viability of HSF cells subsequent to incubation with the formulations compared to cells not subjected to any treatment (control). The results indicated the safety of CUR-Ls at the examined concentrations, maintaining >80% cell viability at all concentrations. In previous research, the components constituting liposomes, such as Tween^®^ 20, oleic acid, PC, and CHO, were determined to be nontoxic [55]. These constituents are frequently utilized in numerous transdermal drug delivery systems; thus, the safety of the formulation has been established [56].

### 2.5. Preparation and Optimization of Hydrogel by DoE

The hydrogels were prepared using the polymer casting method, where the compositions of each hydrogel were altered using the generated experimental runs. Once the input factors (HA (A_1_) and PVA (A_2_)) were varied, the resulting responses were obtained (Table 4). The %swelling (R_1_) ranged from 121% to 213%, the Young’s modulus (R_2_) was 97–146 Pa/%, and the % erosion (R_3_) was between 16% and 37%. An appropriate model for the explanation was selected, and the responses regarding the input factors were predicted based on the assessment of various parameters derived from the regression analysis. ANOVA and multiple regression analyses were performed to establish the model’s significance. Notably, all response *p*-values were <0.05, when the cubic model was fitted, indicating the significance of the model in describing the relationships between the input factors and output responses (Table 5). Moreover, the lack of fit (*p* > 0.05) confirmed the adequacy of the multiple regression model. Accordingly, the R^2^ values for R_1_, R_2_, and R_3_ were 0.8424, 0.9957, and 0.9788, respectively. These coefficients underscored the appropriateness of the predicted models in elucidating strong relationships with values close to 1.000 [38].

These two polymers were selected based on their characteristics and physicochemical properties. HA is a naturally occurring polymer found in the extracellular matrix of connective tissues. The polymer shows great promise for producing wound dressings with the ability to stick to injured tissues due to its viscoelastic and mucoadhesive qualities. Furthermore, HA is critical in the different phases of wound healing as it promotes the migration, proliferation, and stimulation of fibroblast and keratinocyte tissues [57]. Moreover, PVA has several advantages, including nontoxicity, excellent biocompatibility, high biodegradability, and exceptional hydrophilic characteristics. It is commonly used in transdermal applications [58]. However, PVA is limited by its insufficient swelling ability for adequate wound healing [59]. PVA is frequently mixed with other polymers to improve its swelling characteristics. The hydrogel proposed in this study would result from bifunctional cross-linking, namely, physical interactions, e.g., hydrogen bonding and hydrophobic force, combined with the metal coordination complex. Physical interactions were generated using the freeze–thaw technique. Hydrogen bonds are created when the hydroxyl groups in the polymer chain come into contact with water molecules. Crystal growth caused freezing of the polymer solution, which served as a cross-linking point for the polymer chains. The polymer chains became more flexible after thawing. As the freeze–thaw cycle was repeated, numerous crosslink points were created [60]. Conversely, a metal coordination complex was created between aluminum ions acting as coordination centers and the surrounding hydroxyls, carbonyls, and amines. This coordinate covalent bond would strengthen the mechanical characteristics of the hydrogel. Aluminum glycinate was chosen because polyvalent metal ions can form strong complexes during hydrogel formation. Despite its potential toxicity, the skin penetration of aluminum and the concentration used in the formulation were rather low compared to the blood level toxicity (100 µg/L). Thus, aluminum glycinate has been used in cosmetic products as astringents, assuring the dermal safety profile of the compound. Moreover, bifunctional cross-linking would aid CUR-Ls delivery by improving the tolerability of the hydrogel patch against water, moisture, and exudates. During application of wound dressings, water absorption may reduce the toughness of the hydrogel when the patch swells. The multi-mechanism cross-linking would reduce the degradation of the hydrogel patch. This dual-cross-linking mechanism was expected to enrich the structural attributes and toughness and durability of the hydrogels.

**Figure 5:**
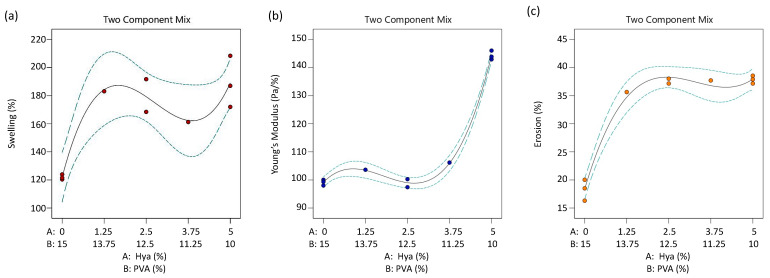
Model graphs illustrating the effect of the hydrogel composition toward (**a**) %swelling, (**b**) Young’s modulus, and (**c**) %erosion.

RSM was employed to investigate the parameter interactions and relationships. Analysis of the regression equation and response surface graphs revealed that the quantities of HA and PVA had a positive influence on all output responses (% swelling, Young’s modulus, and %erosion) (Figure 5). However, the interaction between these factors (A_1_A_2_) resulted in adverse effects on the swelling and mechanical strength, suggesting an increase in these parameters when the polymer quantities were decreased. The model’s optimization predicted an ideal hydrogel formulation comprising 5% HA and 10% PVA with a desirability of 0.41 (Table 6). The lower desirability was due to the ideal response for %erosion being the opposite of the other responses. The combination of HA and PVA at the optimized ratio would yield maximum %swelling and Young’s modulus. While minimizing the %erosion was desired, from the predicted model, the %erosion of the optimized HA:PVA ratio would also be increased. This undesirable prediction led to a desirability value of less than 0.63, which has been reported to be poor. This may be because the hydrogel loosely cross-linked after the freeze–thaw cycles for the hydrogen bond created were not as much compared to the formulas with higher PVA [60]. Conversely, the hydrogel flexibility and swelling capability were reduced when the quantity of polymer was initialized, allowing greater hydrogen bonds to be formed during cycles. Although %erosion would be minimized, the hydrogel properties from this consequence were not expected to be a promising wound healing patch. Moreover, an unacceptable desirability of less than 0.37 was stated to be considered unacceptable. Although the desirability was not ideal, the optimized formula obtained was somewhat acceptable considering the function of the hydrogel patch [43,61]. An optimized hydrogel patch was prepared to ensure optimization by comparing the actual responses with the predicted values (Table 6). It was found that all responses were comparable with the computerized solution, confirming that the selected composition was optimized and reproducible with the desired characteristics.

### 2.6. Addition of the Synthesized PNVP-ITA to the Hydrogel

After the synthesis of PNVP-ITA following the scheme in Figure S1 (ESI†), the obtained polymer was characterized by proton nuclear magnetic resonance spectroscopy (^1^H-NMR) and Fourier transform infrared spectroscopy (FT-IR) to confirm the successful synthesis. The results are presented in Figures S2 (ESI†) and S3 (ESI†), respectively. As shown in the ^1^H-NMR spectra, the peaks at 4.65–4.70 and 6.82 ppm, which were the vinyl groups of NVP, disappeared in the spectrum of PNVP-ITA, demonstrating that the functional group was acquired in the polymerization reaction. Consequently, ITA exhibited peaks at around 3.4, 5.8, and 6.3 ppm, which were attributed to the methylene groups of ITA. After chain propagation, the missing methylene peak in the PNVP-ITA spectrum confirmed polymerization, indicating the successful synthesis of the PNVP-ITA [62]. Considering the FT-IR spectra, CH asymmetric stretching of the polymer chain was observed at 2925 cm^−1^. Strong peaks at 1555 and 1397 cm^−1^ correspond to the symmetric stretching of the carboxylate groups from ITA. O–H stretching from carboxylic acid and hydrogen bonds was observed as a broad band at 3357 cm^−1^. Additionally, the cyclic amide (O=CN) and CN– of NVP were present at 1635 and 1198 cm^−1^, respectively. Thus, the synthesis of PNVP-ITA was confirmed.

**Figure 6:**
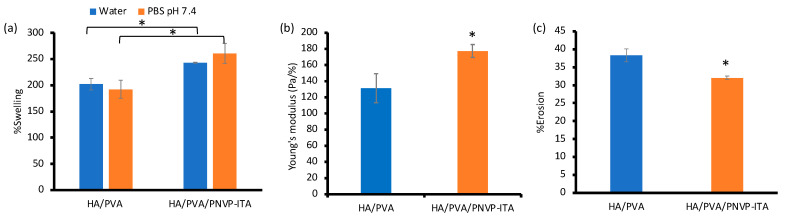
(**a**) %Swelling, (**b**) Young’s modulus, and (**c**) %erosion of HA/PVA/PNVP-ITA hydrogels compared to HA/PVA patches (* Significant difference, *p* < 0.05).

The optimized hydrogel formulation obtained from the computer model was supplemented with 2%wt of the synthesized PNVP-ITA to improve the properties of the patch. Further, the %swelling and Young’s modulus of the HA/PVA/PNVP-ITA improved by 40.4% and 35.0%, respectively (Figure 6), and the %erosion decreased by 6.3%. These findings demonstrate the presence of bifunctional cross-linking among the initiated polymers. PNVP-ITA was synthesized as a carbonyl- and carboxyl-rich polymer intensely aligned for coordination cross-linking. The synthesized PNVP-ITA would allow greater metal coordination bond with Al^3+^ as a cross-linker. Moreover, the polymer can increase the cross-linking points with the larger chains (HA and PVA) during freeze–thaw cycles. Therefore, the degree of cross-linking can be enhanced, resulting in less erosion and a greater Young’s modulus. Additionally, the empowered hydrogel via bifunctional cross-linking facilitated hydrogel swelling, possibly by creating more gaps for trapping water molecules. The formation of this metal coordinate covalent complex improved the physical and mechanical attributes of the hydrogels. This multi-mechanism of cross-linking may enable the delivery of the active compound by maintaining the integrity of the hydrogel structure and resisting erosion upon exudate absorption. When the hydrogel swells, the patch may lose its toughness and strength to hold on to the structure and ability to perform its indication properly [63,64]. Moreover, the swelling capabilities in water and in PBS pH 7.4 were comparable, exemplifying the benefits of applying on the wounds. The addition of the synthesized polymer improved the mechanical properties and degradation of the hydrogel patch, as indicated by the low %erosion. The addition of PNVP-ITA enhanced the properties of the hydrogel and confirmed the dual-cross-linking mechanism of the HA/PVA/PNVP-ITA hydrogel.

### 2.7. Drug Loading and In Vitro Drug Release Study

**Figure 7:**
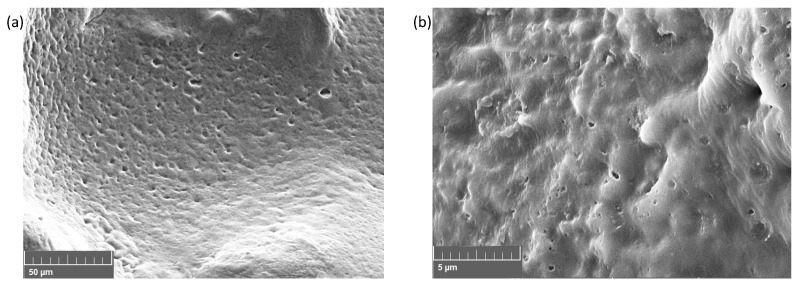
The SEM image of CUR-L@HA/PVA/PNVP-ITA hydrogels at (**a**) 1k× and (**b**) 10k× magnification.

After the preparation of CUR-L@HA/PVA/PNVP-ITA, a photograph of the hydrogel patch was obtained (Figure S4, ESI†). The HA/PVA/PNVP-ITA hydrogel was a homogeneous white patch with 5 mm thickness, while the CUR-L@HA/PVA/PNVP-ITA patch presented a uniform yellow patch of loaded CUR-Ls. In addition, the morphology was assessed using SEM. As presented in Figure 7, the hydrogels were tightly packed, presenting a rather smooth surface. CUR-L was not clearly seen due to the liposome structure embedded within the hydrogel network. Thus, the polymer matrix hindered clear observation of the nanoscaled liposomes. The SEM micrograph was imaged with a TESCAN MIRA3 at 10 kV (1k× and 10k×).

The amount of CUR in the HA/PVA/PNVP-ITA was assayed relative to the expected amount of drug in the hydrogel (0.5% CUR by polymer weight). The absorption method showed that 95.22 ± 4.24% of CUR was absorbed into the hydrogel, which was acceptable considering that almost complete absorption was observed. The patch was cut into a 1.5 × 1.5 cm dimension according to the preliminary experiment to ensure homogeneity of the CUR-L throughout the entire patch after the adsorption of the liposomal drug solution.

**Figure 8:**
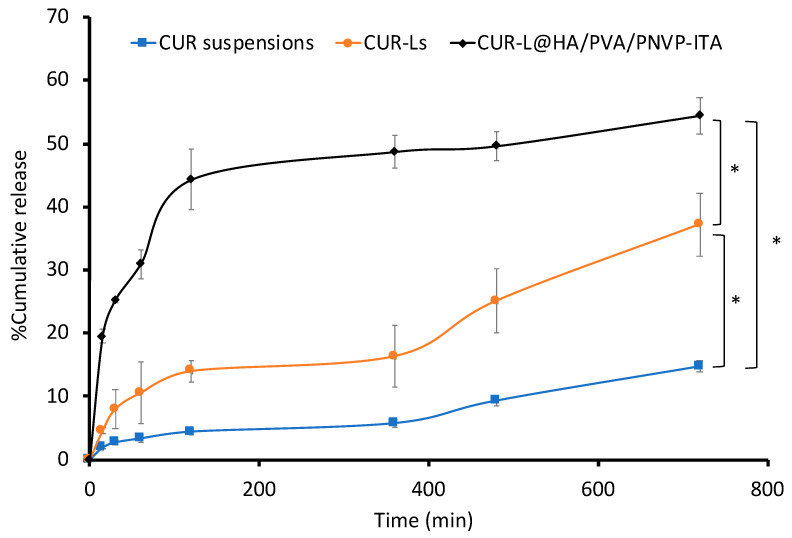
Drug release profile of CUR suspensions (■), CUR-Ls (●), and CUR-L@HA/PVA/PNVP-ITA (◆) (* Significant difference, *p* < 0.05).

The release behavior of CUR from the CUR-Ls and CUR suspensions was compared using the dialysis method. Figure 8 presents a distinct release pattern. CUR-Ls exhibited a more rapid release profile compared to CUR suspensions, which showed a very gradual release into the medium due to its poor solubility. The %cumulative CUR release from CUR-Ls exceeded that of CUR suspensions, possibly due to the improved solubility of CUR. Liposomes exhibit superior features over traditional drug delivery systems, including regulated or prolonged release, protection against drug degradation, increased therapeutic effects, and reduced risk of severe side effects [65]. Note that the maximum release was only observed at 12-h, and further time point was not collected in this study.

The release profiles were fitted to different mathematical models to assess their kinetic profiles. Table 7 shows that the Higuchi model was the most appropriate model for CUR suspension release, and the Korsmeyer-Peppas model offered the best fit for the release of CUR -L from the HA/PVA/PNVP-ITA hydrogel, as evidenced by the highest correlation coefficient (R^2^) [66,67]. Particularly, the release of the CUR suspension showed the highest R^2^ model selection criterion (MSC), indicating a remarkable fit with the Higuchi kinetic model. This implies that diffusion was the major mechanism of CUR release from the suspensions. For the release of CUR-L, while the Korsmeyer-Peppas model may offer the best fit, the Higuchi model provides a more general and simpler explanation of the drug release process. The CUR-L release from the HA/PVA/PNVP-ITA hydrogel showed the highest R^2^ and MSC, indicating a strong fit with the Korsmeyer-Peppas model [68]. An n value of 0.232 indicates a Fickian diffusion-controlled release of CUR from the hydrogel, which is beneficial for achieving controlled and sustained drug release in wound healing applications. The R^2^ and MSC are indicators of goodness-of-fit when comparing multiple models. When comparing multiple models, the R^2^ can be used to distinguish between the most suited models. A higher R^2^ value suggests that the model accurately describes the drug release profile. Additionally, the MSC values can be used to identify the best fit model, as they show how well the model fits the data [69]. A higher MSC value indicates a better model. It balances the fit of the model and the complexity (number of parameters), with higher values suggesting a more reliable and generalizable model. Thus, CUR delivery through CUR-L-embedded hydrogels was effectively described by the Korsmeyer-Peppas model, following a diffusion-controlled release mechanism. However, it should be noted that the release of CUR from the CUR-L@HA/PVA/PNVP-ITA did not require diffusion across the dialysis membrane, which may be the cause of higher drug release.

### 2.8. In Vivo Skin Recovery Study

The wounds were created by surgically removing the epidermis from the mice for the investigation of skin recovery [70]. The wound area was recorded over time, and wound closure was calculated based on the wound size relative to the initial dimensions (percentage of skin recovery).

**Figure 9:**
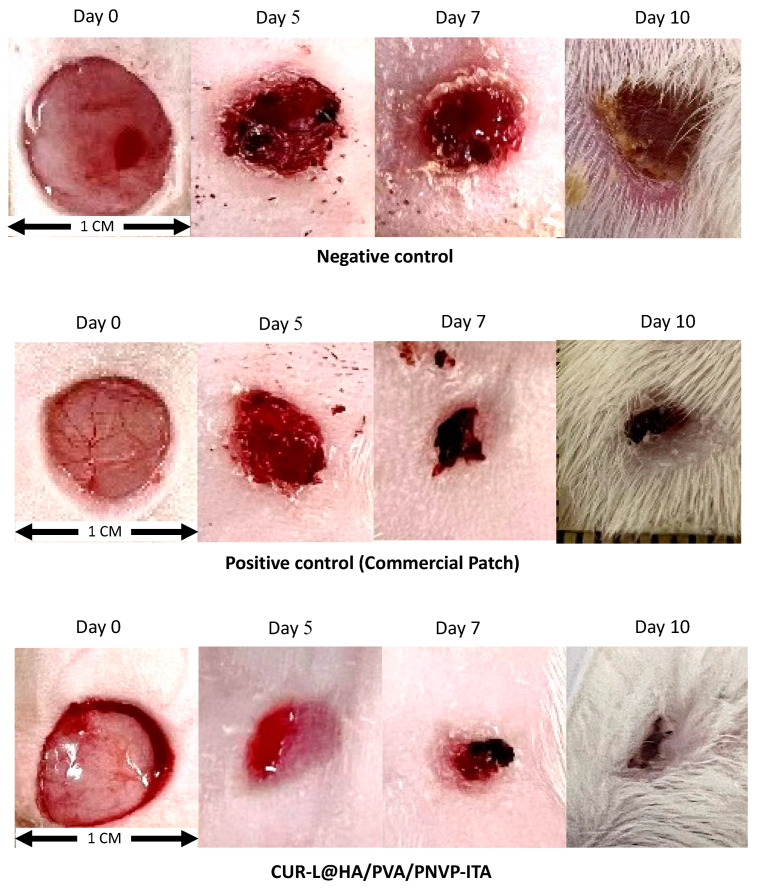
Images of wounds treated with different wound dressings on days 0, 5, and 10.

The results showed that CUR-L@HA/PVA/PNVP-ITA induced a significantly higher percentage of skin recovery than traditional wound dressing at all time points (Table 8, Figure 9). In the negative control group, in which the wounds were covered with sterile gauze, natural wound healing showed the slowest skin recovery rate. The wounds treated with CUR-L@HA/PVA/PNVP-ITA showed greater wound closure than the control wounds. Hence, the developed formulation showed a faster wound healing rate compared to a commercial hydrogel patch, with a significantly higher percentage of skin recovery on day 5. The CUR-L@HA/PVA/PNVP-ITA patch presented a more complete wound closure on day 10 compared to the commercial patch, which showed no difference compared to the wound closure on day 7. This was possible because CUR induces inflammatory cell apoptosis during the early phase of wound healing. This compound can also accelerate the healing process by shortening the inflammatory phase [28]. Additionally, CUR has been shown to increase TGF-β1 formation in wounds, stimulate fibroblast growth, improve integrin receptor expression, and facilitate cell migration to the wound site. Furthermore, its antioxidant properties are essential for wound care [71]. With the increased antioxidant activity of CUR-Ls, the antioxidative property could play a role in improving the wound healing effect, leading to more rapid skin recovery. CUR has also been reported to have an intrinsic antibacterial effect against various strains, for example, *Staphylococcus aureus*, *Escherichia coli*, *Enterococcus faecalis*, and *Pseudomonas aeruginosa*, which may cause wound infections. This effect could also contribute to creating a favorable environment for wound healing [72]. Moreover, the application of hydrogel patches may have facilitated wound healing by providing a moist environment to the wound, allowing hydration and improving the healing process. Moreover, the hydrogels absorb wound exudates, creating a more favorable healing environment and protecting the wound from external harm [73]. Thus, the bifunctional cross-linking mechanism provided a rigid hydrogel patch throughout the examination, despite water absorption. These findings indicate the possibility of using CUR-L@HA/PVA/PNVP-ITA as a wound dressing. The efficacy and enhanced wound healing rate of the prepared formulation were considered when CUR-L was embedded into the HA/PVA/PNVP-ITA hydrogel, whereas the effect of the blank hydrogel or CUR-L was not established.

The effect of CUR formulation on wound healing has been established in different hydrogel platforms and investigated in different aspects. Maryam Khaleghi et al. (2023) developed CUR-loaded HA hydrogel, which showed high potential to heal the cutaneous wounds on the mouse excisional wound model by repairing histopathological damages rapidly and without a scar. Due to the effect of both CUR and HA, the combination served as a multipotent biomaterial for medical applications regarding the treatment of chronic, infected, and dehiscent wounds [74]. Chopra et. al. (2023) examined the effect of chitosan–PVA hydrogel films loaded with CUR. The hydrogel matched the characteristics required for wound healing. The hydrogel film presented significant antimicrobial activity, while in silico studies showed good binding energy scores among CUR and key wound healing residues of inflammatory proteins [75]. Ngoc Le et al. (2023) investigated the wound healing potential of CUR-loaded nanoemulsion-based gel. The formulation had superior efficacy than that of the CUR gel and commercial gel. Tissue regeneration in epithelial and dermal tissues was more considerable, specifically in terms of histopathological changes. Their gel formulation improved re-epithelization and vascularization in injured tissue, thereby accelerating wound healing [76]. Above all, the use of CUR-embedded hydrogel presented significant improvement in wound healing efficacy. This study revealed the optimization of the CUR-Ls and HA/PVA hydrogels with their combined effect on wound healing in vivo. Thus, the HA played a proficient role in cell proliferation and angiogenesis; PVA presented a promising platform for drug delivery, while CUR regulated and facilitated wound healing effects in combination with the developed platform.

## 3. Conclusions

This study demonstrated the effectiveness of the CUR-L@HA/PVA/PNVP-ITA hydrogel in improving wound healing. The liposomes were optimized using a statistical computer program to achieve a small particle size with a suitable ZP and maximized CUR content. The optimized liposomes showed better antioxidant activity than the CUR suspensions, with comparable safety. HA/PVA/PNVP-ITA hydrogels with promising physical and mechanical properties were created through the design of the experiment. The incorporation of CUR into liposomes enhanced its release, presumably as a result of increased solubility. CUR release from the nanocomposite hydrogel followed the Korsmeyer−Peppas model, indicating a Fickian diffusion-controlled mechanism. Furthermore, in vivo experiments revealed that the CUR-L@HA/PVA/PNVP-ITA hydrogel accelerated skin healing. Most importantly, the developed CUR-L@HA/PVA/PNVP-ITA hydrogel is an appropriate method for CUR delivery in wound healing. The improved solubility of CUR and its swellable and durable characteristics were the key features of the prepared formulation for wound healing. The computer-aided approach allowed liposomes and hydrogel formulations to present the desired characteristics, specifically for wound healing applications. These formulations may be further developed for other applications by tuning the optimization criteria, giving the chance to establish a wider range of optimal formulations. Hence, CUR-L@HA/PVA/PNVP-ITA hydrogel could be a favorable candidate after further clinical evaluations.

## 4. Materials and Methods

### 4.1. Materials

CUR, Tween^®^ 20, oleic acid, soybean phosphatidylcholine (PC), cholesterol (CHO), 2,2-diphenyl-1-picrylhydrazyl (DPPH), ascorbic acid, methyl-thiazolyl diphenyl tetrazolium bromide (MTT), polyethylene glycol (PEG) 400, dimethyl sulfoxide (DMSO), PVA, itaconic acid (ITA), *N*-vinylpyrrolidone (NVP), and 2,2′-azobis(2-methylpropionamidine) dihydrochloride () were purchased from Sigma Chemicals (St. Louis, MO, USA). Chloroform and methanol were acquired from Merck & Co. (Darmstadt, Germany). Hyaluronic acid (HA, MW = 1550 kDa) was procured from P.C. Chemicals (Bangkok, Thailand). Penicillin-streptomycin, fetal bovine serum (FBS), and Dulbecco’s modified Eagle’s medium (DMEM) were bought from Gibco BRL (Rockville, MD, USA). Human skin fibroblast (HSF) cells were procured from the American Type Culture Collection (Rockville, MD, USA). Other chemical substances and solvents were used as obtained.

### 4.2. Preparation and Optimization of Curcumin-Loaded Liposomes by DoE

The design of the experiment (DoE) methodology aids in understanding the impact of variables and their interrelationships with the responses. The selected independent variables, including the quantity of CUR, Tween^®^ 20, and oleic acid, were varied by the Box–Behnken experimental design. The particle size, polydispersity index (PDI), zeta potential (ZP), and CUR content were the dependent factors. The response surface methodology (RSM) was utilized along with a regression equation to determine the correlation between the independent and dependent variables. The outcomes were analyzed using Design Expert^®^ 11 software (Stat-Ease, Minneapolis, MN, USA). The significance level was set at a 95% confidence interval, with a *p*-value of 0.05.

CUR-Ls were prepared using the thin-film hydration method. The liposomes were formed using 50 mM PC and 50 mM CHO at a 10:2 molar ratio, with different concentrations of Tween^®^ 20, oleic acid, and CUR (Table 1). Initially, CUR was dissolved in PC and CHO in a solvent mixture of chloroform and methanol (2:1). The resultant solution was subjected to evaporation under nitrogen gas flow to create a thin film and desiccated for 24 h. Subsequently, Tween^®^ 20 and oleic acid as edge activators were added to phosphate-buffered solution (PBS) pH 7.4, followed by vortex mixing and particle size reduction using a probe sonicator (Vibra-Cell^TM^, Sonics, and Materials, CT, USA) for 30 min. Then, centrifugation was conducted at 12,000 rpm and 4 °C for 30 min. Afterward, the supernatants were collected for further analysis.

### 4.3. Characterizations of Curcumin-Loaded Liposomes

#### 4.3.1. Particle Size, PDI, and ZP

The particle size, PDI, and ZP of the liposomes were evaluated with the dynamic light scattering technique using the Zetasizer Nano-ZS (Malvern, Worcestershire, UK) at a 90° angle and 25 °C. The samples were measured in triplicate immediately after the preparation.

#### 4.3.2. Drug Determination

CUR within the CUR-Ls was quantified using a direct content determination method [54,77]. Methanol was employed to disrupt the liposomes prior to a 200-fold dilution. High-performance liquid chromatography (HPLC; Agilent 1100 Series, Santa Clara, CA, USA) was utilized with a C18 column (250 mm × 4.6 mm, 5 µm) maintained at 33 °C. An injection volume of 20 μL was employed. The isocratic mobile phase was composed of acetonitrile and 2% *w*/*v* acetic acid (50:50). The compound was analyzed using a UV detector at 425 nm. The flow rate was fixed at 1.2 mL/min. A standard curve of CUR concentrations at 1–100 μg/mL with a correlation coefficient of 0.999 was prepared for drug content analysis.

### 4.4. Differential Scanning Calorimetry

A differential scanning calorimeter (DSC) (Sapphire, PerkinElmer, Waltham, MA, USA) was utilized to examine the thermal characteristics of CUR in CUR-Ls. Around 3 mg of the sample was placed in a hermetically sealed aluminum pan. The analysis was performed over a temperature range from 25 to 300 °C heated at a constant rate of 10 °C per minute, under a steady nitrogen flow.

### 4.5. Antioxidant Activity

The ability of CUR to scavenge free radicals was tested using the DPPH radical scavenging assay, which was performed by preparing samples (CUR suspensions or CUR-Ls) at different concentrations in PBS. The CUR suspension was subjected to bath sonication for 2 h to allow maximum dissolution. DPPH solution was prepared in methanol and subsequently added to the samples (1:1 *v*/*v*). A standard curve was prepared using ascorbic acid as a positive control at 1–1000 μg/mL, with a correlation coefficient of 0.999. The reaction mixture was shaken slightly and left in the dark at room temperature for 30 min. Then, the absorbance of the mixture was measured spectrophotometrically at 515 nm to determine the percentage of DPPH radical scavenging using Equation (1):

(1) $$\%\mathrm{DPPH}\;\mathrm{radical}\;\mathrm{scavenging}\;=\frac{A_{\mathrm{sample}\;}-A_{\mathrm{blank}\;}}{A_{\mathrm{control}\;}-A_{\mathrm{blank}\;}}\;\times 100$$

where $A_{\mathrm{control}\;}$ is the absorbance of the control (DPPH solution in methanol), $A_{\mathrm{sample}\;}$ is the absorbance of the sample (sample with DPPH), and $A_{\mathrm{blank}\;}$ is the absorbance of the blank (sample without DPPH).

### 4.6. Cytotoxicity Assessment

The potential cytotoxic effects of the CUR-Ls were evaluated through an MTT assay. HSF cells were cultured in serum-supplemented DMEM and seeded in a 96-well plate at a density of 10,000 cells/well. The cells were cultivated at 37 °C in 95% air and 5% CO_2_ until they reached 80% confluence. Afterward, the cells were exposed to CUR or CUR-Ls in serum-free DMEM prepared at 0.1–100 µg/mL concentrations for 24 h. After exposure, the cells were rinsed with sterile PBS pH 7.4 and treated with 100 µL of 1 mg/mL MTT solution in FBS-supplemented DMEM. The formazan precipitate formed during a 3 h incubation was dissolved in 100 µL of DMSO, and the absorbance was measured at 550 nm (VICTOR Nivo^TM^, Perkin Elmer, MA, USA). Finally, the %relative cell viability was calculated using Equation (2):

(2) $$\mathrm{Relative}\;\mathrm{cell}\;\mathrm{viability}\;\left(\%\right)=\frac{{\mathrm{Abs}}_{550,\;\mathrm{sample}\;}-\;{\mathrm{Abs}}_{550,\;\mathrm{blank}\;}}{{\mathrm{Abs}}_{550,\;\mathrm{control}}\;-\;{\mathrm{Abs}}_{550,\;\mathrm{blank}\;}}\times 100$$

### 4.7. Synthesis of Poly(N-Vinylpyrrolidone-Co-Itaconic Acid)

PNVP-ITA was synthesized using a surfactant-free emulsion polymerization reaction. PNVP-ITA was synthesized to yield a polymer with carboxylic acids to improve the cross-linking density of the post-optimization hydrogels. Therefore, V50 (2%wt) as a radical polymerization initiator was added to 100 mL deionized water heated at 75 °C with nitrogen flush to provide an inert atmosphere. Moreover, NVP (10 mmol) was mixed with ITA (30 mmol) in 10 mL chloroform. The monomer mixture was slowly introduced into the warmed initiator solution under an inert atmosphere and allowed to polymerize for at least 18 h. Then, the reactant was left to cool down and purified via dialysis (MW cutoff, 3.5 kDa) against deionized water for 3 days (fresh water replenished every 6 h). The dried polymer was collected after lyophilization. The synthesized polymers are described in the electronic supplementary information (ESI†).

The synthesized PNVP-ITA was characterized using ^1^H-Nuclear magnetic resonance spectroscopy (NMR) and Attenuated Total Reflectance-Fourier-transform infrared spectroscopy (ATR-FTIR). The ^1^H-NMR spectra were collected with a 300 MHz (AVANCE III HD, Bruker) spectrometer at 298 K. All chemical shifts were reported as δ in parts per million (ppm), using the chemical shift of D_2_O (δ = 4.80 ppm) as a reference. The chemical structure of PNVP-ITA was confirmed by an ATR-FTIR spectrophotometer (Nicolet iS5, Thermo Fisher Scientific, Bedford, MA, USA). The spectrum with wavenumber ranging from 4000 to 500 cm^−1^ was collected with a resolution of 4 cm^−1^ and a total of 32 scans/run.

### 4.8. Preparation and Optimization of Hydrogel by DoE

The central composite design was utilized to determine the impact of independent variables, specifically the HA (X_1_) and PVA (X_2_) concentrations used to form the hydrogels. The dependent variables were water absorption (Y_1_), percentage of erosion (Y_2_), and mechanical properties (Y_3_). RSM, along with a regression equation, was used to investigate the relationship between independent and dependent variables. All experimental results were analyzed using Design Expert^®^ 11 software.

The PVA solution (20%*w*/*v*) was prepared by dispersing the PVA powder in hot deionized water (80 °C) and stirred until homogeneous. The PVA solution was mixed with HA at specific percentages (Table 4). Further, aluminum glycinate (0.2%) and malic acid (0.1%) were obtained as the cross-linker and cross-linking regulators, respectively. Malic acid was added to the polymer mixture and aluminum glycinate was dispersed in 30% glycerin. Then, the glycerin dispersion was added to the polymer mixture and gently mixed until homogeneous. The mixture (10 g) was slowly poured into an acrylic mold to avoid air bubbles in the patch. These polymer mixtures were subjected to 3.86 freeze–thaw cycles. A cycle of freezing at −20 °C for 18 h followed by thawing at room temperature for 6 h was conducted, and 0.86 cycles at 15.48 h of freezing and 5.16 h of thawing [78].

The optimized hydrogel patch was added with 2 wt% PNVP-ITA to improve the properties of the hydrogel patch.

### 4.9. Characterizations of HA/PVA Hydrogels

#### 4.9.1. Water Absorption

The equilibrium or gravimetric analysis method was used to determine the proportion of dissolution media absorbed by the hydrogels, thereby determining the capability of the hydrogel to hold water. Then, 1 g of the hydrogel was cut, accurately weighed (W_0_), and soaked in 5 mL of deionized water at 37 ± 0.5 °C. The sample was removed from the medium after 24 h and lightly wiped with tissue paper to eliminate excess test liquid before being reweighed (W_i_) on an analytical balance (model AG204, Mettler-Toledo, Switzerland). In addition, the water absorption capability of the optimized patch was confirmed by soaking it in PBS pH 7.4 to illustrate the swelling capability under wound conditions. Equation (3) was used to calculate the percentage of water absorption relative to the initial patch weight, as follows:

(3) $$\%Water\;absorption=\frac{W_{i}-\;W_{0}}{W_{0}}\;\times \;100$$

#### 4.9.2. Erosion

Hydrogel erosion was studied to determine the cross-linking density of the patch. Each hydrogel was dried in an oven at 60 °C until a constant weight was obtained (W_0_). Then, the patch was swollen in 25 mL of deionized water for 24 h and dried under the same conditions. The final dried hydrogel was reweighed (W_i_). The percentage of erosion was computed using Equation (4) as follows:

(4) $$\%Erosion=\frac{W_{0}-W_{i}}{W_{0}}\;\times \;100$$

#### 4.9.3. Mechanical Properties

The mechanical strength of the hydrogels was examined using a texture analyzer (TA.XT plus, Stable Micro Systems, Surrey, UK) with a 5 kg load cell. The hydrogel samples were cut to 1 × 5 cm (w × h). Tensile strength was measured using tensile grips, which were set to travel at a constant test speed of 5.0 mm/s until the hydrogel patches broke. The Young’s modulus of the patch was obtained using texture analyzer software.

### 4.10. Preparation of Curcumin Liposome-Embedded Hydrogels

The optimized CUR-Ls were embedded in the HA/PVA/PNVP-ITA hydrogel by absorption. By that, the blank HA/PVA/PNVP-ITA hydrogel patch was cut to 1.5 × 1.5 cm and accurately weighed. Then, the patches were placed in a well plate containing CUR-L solution (equivalent to 0.5% *w*/*w* CUR) and allowed to absorb for 24 h. Thereafter, excess test liquid was carefully removed using tissue paper. The amount of CUR in the hydrogel was determined by cutting the CUR-L@ HA/PVA/PNVP-ITA into small pieces, and CUR was extracted in 5 mL methanol in a tube rotator for 24 h. The total CUR content was measured by HPLC.

### 4.11. Morphology

The morphology of the prepared CUR-L@ HA/PVA/PNVP-ITA was examined under a scanning electron microscope (SEM; Tescan Mira3, Brno, Czech Republic). Swollen hydrogel samples were freeze-dried and then cut into transverse sections before being sputter-coated with gold on a metal stub for SEM scrutinization.

### 4.12. In Vitro Drug Release Study

The release behavior of the CUR solution, CUR-Ls, and CUR-L@ HA/PVA/PNVP-ITA hydrogel was examined using the dialysis method (n = 3). First, 1 mL of the sample solution was enclosed in a dialysis bag (CelluSep^®^ T2, TX, USA; MW cutoff, 6–8 kDa). The bag was placed in a glass bottle containing 10 mL of 20% PEG 400 in PBS (pH 7.4) to establish the appropriate sink conditions [79]. The experiment was conducted at a temperature of 32 °C ± 2 °C inside a shaking incubator agitated at 100 rpm. At 0, 0.25, 0.5, 1, 2, 6, 8, and 12 h intervals, a 1 mL aliquot of the release medium was withdrawn and replaced with an equal volume of fresh medium. CUR content was quantified using HPLC to calculate the cumulative drug release. Notably, the CUR-L@ HA/PVA/PNVP-ITA hydrogel (1 × 1 cm^2^), which was expected to contain 175 µg of CUR, was examined without using a dialysis bag. The amount of CUR in the patch from other randomly selected areas was quantified so that CUR solution and CUR-Ls samples could be prepared at an equivalent initial CUR content.

### 4.13. In Vivo Skin Recovery Study

The animal protocol (ID project no. 2/2566; 23 March 2023) was approved by the institutional animal ethics committee of Silpakorn University, Nakhon Pathom, Thailand. All animal procedures adhered to the National Research Council Guide for the Care and Use of Laboratory Animals. Male 7-week-old Wistar rats, weighing 310–360 g, were procured from the National Laboratory Animal Center, Mahidol University, Nakhon Pathom, Thailand. The rats were housed independently in polypropylene cages with wire mesh covers and randomly divided into three groups:

Group I received a gauze bandage (negative control, n = 6).

Group II received a commercial wound healing patch (positive control, n = 6).

Group III was administered a CUR-L-embedded HA/PVA/ PNVP-ITA hydrogel patch (n = 6).

The rats were caged under controlled room conditions set at a temperature of 25 ± 2 °C, with a relative humidity of 60 ± 10%, and a 12/12-h light–dark cycle. A typical laboratory diet was fed, and unrestricted access to water was provided (082G/15, National Laboratory Animal Center, Mahidol University, Nakhon Pathom, Thailand). A 10-mm-diameter circular wound was created with sterile scissors on the dorsothoracic region of the rat (one incision on the left and one on the right) under proper anesthesia induced by intraperitoneal administration of pentobarbital (50 mg/kg). All sample patches sized 1.5 × 1.5 cm were terminally sterilized using UV light for 15 min per side before testing. In group III, the patches were applied to the wounds and covered with Tegaderm™ occlusive hydrogel film to prevent displacement during the experiment. Wound appearance and size were monitored along with hydrogel patch changes to calculate the percentage of skin recovery (Equation (5)) at days 0, 3, 5, 7, and 10 while the rats were under anesthesia:

(5) $$\%\;\mathrm{Skin}\;\mathrm{recovery}=\frac{A_{0}-A_{t}}{A_{0}}\;\times \;100$$

where A_0_ is the wound area at day 0 and A_t_ is the wound area at day t.

### 4.14. Data Analysis

Each experiment was conducted in triplicate, and the results are expressed as mean ± standard deviation. To compare the two groups, an independent two-sided *t*-test was employed. For statistical analyses involving multiple groups, a one-way analysis of variance test with a post hoc Tukey’s test was performed. SPSS^®^ software version 19 (SPSS Inc., Chicago, IL, USA) was utilized for these analyses. Statistical significance was set at *p* < 0.05.

## Figures and Tables

**Table 1:** Experimental runs generated by Box–Behnken experimental design upon giving the input factors and output responses.

Formulation	Input Factors (X)			Output Responses (Y)			
	X_1_: Tween^®^ 20 (%)	X_2_: Oleic Acid (%)	X_3_: CUR (%)	Y_1_: Size (nm)	Y_2_: PDI	Y_3_: ZP (mV)	Y_4_: CUR Content (µg/mg)
L1	1.5	0.75	5.5	86.61	0.2	−35.7	6.6
L2	0	0.75	10	114.1	0.39	−17.9	1.9
L3	1.5	0.75	5.5	87.2	0.18	−37.3	11.2
L4	3	1.5	5.5	80.2	0.14	−34.7	10.8
L5	0	0	5.5	78.1	0.15	7.3	10.4
L6	0	0.75	1	96.9	0.2	−25.3	2.1
L7	1.5	1.5	1	99.5	0.16	−45	3.5
L8	1.5	1.5	10	110	0.17	−39.7	7.2
L9	3	0	5.5	58.2	0.01	−12.4	17.6
L10	3	0.75	1	63	0.25	−30.5	3.4
L11	1.5	0.75	5.5	75.1	0.2	−35.7	14
L12	1.5	0.75	5.5	88.6	0.2	−38.6	11.7
L13	1.5	0.75	5.5	76.7	0.22	−34	10.1
L14	3	0.75	10	61.2	0.22	−32.4	17
L15	1.5	0	1	64.4	0.17	−9.2	5.9
L16	1.5	0	10	73.2	0.21	−7.57	21.8
L17	0	1.5	5.5	112.6	0.21	−40.3	2

**Table 2:** Summary of the ANOVA and multiple regression analysis for Behnken experimental design.

Responses	Model	*p*-Value	Lack of Fit	R^2^
Particle size	Linear	<0.0001	0.5558	0.8750
PDI	Quadratic	0.0093	0.0106	0.7161
Zeta potential	Quadratic	<0.0001	0.6991	0.9885
CUR content	Linear	0.0012	0.2322	0.6240
Multiple regression equation model (coded equation)				
Y_1_ = 83.86 − 17.39X_1_ + 16.05X_2_ + 4.34X_3_ Y_2_ = 0.2032 − 0.0413X_1_ + 0.0175X_2_ + 0.0262X_3_ − 0.0550X_1_X_3_ − 0.0796X_2_^2^ + 0.0579X_3_^2^ Y_3_ = −36.26 − 4.23X_1_ − 17.23X_2_ + 1.55X_3_ + 6.32X_1_X_2_ − 2.32X_1_X_3_ + 0.9175X_2_X_3_ + 7.54X_1_^2^ − 8.70X_2_^2^ + 2.20X_3_^2^ Y_4_ = 10.72 + 4.05X_1_ − 4.03X_2_ + 4.12X_3_				

**Table 3:** Criteria, solution, and confirmation of CUR-L optimization.

Variables	Goal	Solution	Desirability	Confirmation	*p*-Value
Tween^®^ 20	in range	2.718	1.000		
Oleic acid	in range	0.041			
CUR	in range	8.116			
Size	minimize	55.80 ± 6.35		64.02 ± 3.37	0.1187
PDI	none	0.082 ± 0.003		0.107 ± 0.026	0.2262
Zeta potential	in range	−15.03 ± 1.56		−17.26 ± 3.56	0.1979
CUR content	maximize	22.09 ± 2.22		19.92 ± 0.54	0.1758

**Table 4:** Experimental runs generated by central composite design upon giving the input factors and output responses.

Formulation	Input Factors (A)		Output Responses (R)		
	A_1_: HA (%)	A_2_: PVA (%)	R_1_: Swelling (%)	R_2_: Young’s Modulus (Pa/%)	R_3_: Erosion (%)
H1	2.5	12.5	168.45	97.43	38.02
H2	2.5	12.5	191.72	100.28	37.16
H3	5	10	208.37	146.03	38.55
H4	0	15	123.93	100.06	20.03
H5	0	15	120.39	98.02	16.31
H6	5	10	172.02	143.87	37.16
H7	1.25	13.75	213.11	103.63	35.64
H8	3.75	11.25	161.23	106.19	37.71
H9	5	10	187.00	142.91	37.90
H10	0	15	121.55	99.35	18.51

**Table 5:** Summary of the ANOVA and multiple regression analysis for central composite design.

Responses	Model	*p*-Value	Lack of Fit	R^2^
%Swelling	Cubic	0.0241	0.8901	0.8424
Young’s modulus	Cubic	<0.0001	0.8076	0.9957
%Erosion	Cubic	0.0083	0.2588	0.9788
Multiple regression equation model (coded equation)				
%Swelling = 189.03A_1_ + 121.86A_2_ + 95.18A_1_A_2_ − 295.83A_1_A_2_ (A_1_ − A_2_) Young’s modulus = 144.29A_1_ + 99.16A_2_ − 90.84A_1_A_2_ − 106.25A_1_A_2_(A_1_ − A_2_) %Erosion = 37.95A_1_ + 18.36A_2_ + 40.52A_1_A_2_ − 41.18A_1_A_2_(A_1_ − A_2_)				

**Table 6:** The criteria for the optimization of HA/PVA hydrogel.

Variables	Goal	Solution	Desirability	Confirmation	*p*-Value
HA	in range	5	0.41		
PVA	in range	10			
Swelling	maximize	177.9 ± 12.6		176.2 ± 12.0	0.8731
Young’s modulus	maximize	133.6 ± 14		131.0 ± 18.0	0.8297
Erosion	minimize	38.3 ± 1.4		38.3 ± 1.8	0.9850

**Table 7:** Kinetic models of the CUR suspensions, the CUR-loaded liposomes, and CUR liposomes-embedded hydrogels.

Kinetic Model	Parameter	CUR Suspensions	CUR-Ls	CUR-L @HA/PVA/PNVP-ITA Hydrogels
Zero-order	R^2^	0.8595	0.7688	0.8682
	MSC	1.6768	1.1787	1.2668
	Equation	F = 0.02t	F = 0.053t	F = 0.099t
First-order	R^2^	0.8610	0.7908	0.9139
	MSC	1.6876	1.2789	0.9367
	Equation	$F=100(1-\;e^{-0.0002t})$	$F=100(1-\;e^{-0.001t}$)	$F=100(1-e^{-0.002t}$)
Higuchi	R^2^	0.8762	0.9280	0.9186
	MSC	1.8035	2.1456	0.1710
	Equation	$F=0.451\sqrt{t}$	$F=1.208\sqrt{t}$	$F=2.472\sqrt{t}$
Krosmeyer-Peppas	R^2^	0.8219	0.9649	0.9850
	MSC	1.8019	1.9199	2.3622
	Equation	F = 0.162t^n−1^	F = 0.957t^n−1^	F = 12.191t^n−1^
	n	0.668	0.538	0.232

where F is the percentage of released curcumin in time, t (min).

**Table 8:** Percentage of skin recovery after applying the patches on days 3, 5, 7, and 10 (Significant difference compared to negative control (*) and positive control (^#^), *p* < 0.05.

	Day 0	Day 3	Day 5	Day 7	Day 10
Negative control	0.0 ± 0.0	30.8 ± 4.2	46.2 ± 4.1	53.8 ± 5.6	65.1 ± 5.4
Commercial Patch	0.0 ± 0.0	27.3 ± 8.0	45.5 ± 3.9	81.8 ± 6.5 *	84.3 ± 6.3 *
CUR-L@HA/PVA/PNVP-ITA	0.0 ± 0.0	25.0 ± 5.0	66.0 ± 5.6 *^,#^	88.3 ± 2.1 *	95.2 ± 2.1 *^,#^

## Data Availability

Data available on request due to restriction.

## Supplementary Materials

The following supporting information can be downloaded at: https://www.mdpi.com/article/10.3390/gels10090598/s1, Figure S1: Synthesis scheme of PNVP-ITA; Figure S2: ^1^H-NMR spectra of NVP, ITA, PNVP-ITA; Figure S3: ATR-FTIR spectrum of PNVP-ITA; Figure S4: The photograph of (a) blank HA/PVA/PNVP-ITA hydrogel and (b) CUR-L@HA/PVA/PNVP-ITA hydrogels.

## References

[1] Las Heras K., Igartua M., Santos-Vizcaino E., Hernandez R.M. (2020). Chronic wounds: Current status, available strategies and emerging therapeutic solutions. J. Control Release.

[2] Sen C.K. (2023). Human Wound and Its Burden: Updated 2022 Compendium of Estimates. Adv. Wound Care.

[3] Boateng J.S., Matthews K.H., Stevens H.N., Eccleston G.M. (2008). Wound healing dressings and drug delivery systems: A review. J. Pharm. Sci..

[4] Kumar A., Jaiswal M. (2015). Design and in vitro investigation of nanocomposite hydrogel based in situ spray dressing for chronic wounds and synthesis of silver nanoparticles using green chemistry. J. Appl. Polym. Sci..

[5] Augustine R., Augustine A., Kalarikkal N., Thomas S. (2016). Fabrication and characterization of biosilver nanoparticles loaded calcium pectinate nano-micro dual-porous antibacterial wound dressings. Prog. Biomater..

[6] Aljghami M.E., Saboor S., Amini-Nik S. (2019). Emerging Innovative Wound Dressings. Ann. Biomed. Eng..

[7] Abruzzo A., Cappadone C., Sallustio V., Picone G., Rossi M., Nicoletta F.P., Luppi B., Bigucci F., Cerchiara T. (2021). Development of Spanish Broom and Flax Dressings with Glycyrrhetinic Acid-Loaded Films for Wound Healing: Characterization and Evaluation of Biological Properties. Pharmaceutics.

[8] Gefen A., Alves P., Beeckman D., Lazaro-Martinez J.L., Lev-Tov H., Najafi B., Swanson T., Woo K. (2023). Mechanical and contact characteristics of foam materials within wound dressings: Theoretical and practical considerations in treatment. Int. Wound J..

[9] Van Vlierberghe S., Dubruel P., Schacht E. (2011). Biopolymer-based hydrogels as scaffolds for tissue engineering applications: A review. Biomacromolecules.

[10] Bilici Ç., Can V., Nöchel U., Behl M., Lendlein A., Okay O. (2016). Melt-processable shape-memory hydrogels with self-healing ability of high mechanical strength. Macromolecules.

[11] Singh B., Sharma S., Dhiman A. (2013). Design of antibiotic containing hydrogel wound dressings: Biomedical properties and histological study of wound healing. Int. J. Pharm..

[12] Su J., Li J., Liang J., Zhang K., Li J. (2021). Hydrogel preparation methods and biomaterials for wound dressing. Life.

[13] Iranmanesh P., Ehsani A., Khademi A., Asefnejad A., Shahriari S., Soleimani M., Ghadiri Nejad M., Saber-Samandari S., Khandan A. (2022). Application of 3D Bioprinters for Dental Pulp Regeneration and Tissue Engineering (*Porous architecture*). Transp. Porous Media.

[14] Chen Y., Li J., Lu J., Ding M., Chen Y. (2022). Synthesis and properties of Poly(vinyl alcohol) hydrogels with high strength and toughness. Polym. Test..

[15] Chopra H., Bibi S., Kumar S., Khan M.S., Kumar P., Singh I. (2022). Preparation and Evaluation of Chitosan/PVA Based Hydrogel Films Loaded with Honey for Wound Healing Application. Gels.

[16] Zhong Y., Lin Q., Yu H., Shao L., Cui X., Pang Q., Zhu Y., Hou R. (2024). Construction methods and biomedical applications of PVA-based hydrogels. Front. Chem..

[17] Wang D., Cui F., Xi L., Tan X., Li J., Li T. (2023). Preparation of a multifunctional non-stick tamarind polysaccharide-polyvinyl alcohol hydrogel immobilized with a quorum quenching enzyme for maintaining fish freshness. Carbohydr. Polym..

[18] Li M., Chen D., Sun X., Xu Z., Yang Y., Song Y., Jiang F. (2022). An environmentally tolerant, highly stable, cellulose nanofiber-reinforced, conductive hydrogel multifunctional sensor. Carbohydr. Polym..

[19] Liang H., Mirinejad M.S., Asefnejad A., Baharifar H., Li X., Saber-Samandari S., Toghraie D., Khandan A. (2022). Fabrication of tragacanthin gum-carboxymethyl chitosan bio-nanocomposite wound dressing with silver-titanium nanoparticles using freeze-drying method. Mater. Chem. Phys..

[20] Li L., Zhang Y., Lu H., Wang Y., Xu J., Zhu J., Zhang C., Liu T. (2020). Cryopolymerization enables anisotropic polyaniline hybrid hydrogels with superelasticity and highly deformation-tolerant electrochemical energy storage. Nat. Commun..

[21] Liu L., Zhu M., Xu X., Li X., Ma Z., Jiang Z., Pich A., Wang H., Song P. (2021). Dynamic Nanoconfinement Enabled Highly Stretchable and Supratough Polymeric Materials with Desirable Healability and Biocompatibility. Adv. Mater..

[22] Luo Z., Wang Y., Li J., Wang J., Yu Y., Zhao Y. (2023). Tailoring Hyaluronic Acid Hydrogels for Biomedical Applications. Adv. Funct. Mater..

[23] Parhi R. (2017). Cross-Linked Hydrogel for Pharmaceutical Applications: A Review. Adv. Pharm. Bull..

[24] Xue X., Hu Y., Wang S., Chen X., Jiang Y., Su J. (2022). Fabrication of physical and chemical crosslinked hydrogels for bone tissue engineering. Bioact. Mater..

[25] Tabasi H., Babaei M., Abnous K., Taghdisi S.M., Saljooghi A.S., Ramezani M., Alibolandi M. (2021). Metal–polymer-coordinated complexes as potential nanovehicles for drug delivery. J. Nanostructure Chem..

[26] Liu L., Sun L., Wu Q., Guo W., Li L., Chen Y., Li Y., Gong C., Qian Z., Wei Y. (2013). Curcumin loaded polymeric micelles inhibit breast tumor growth and spontaneous pulmonary metastasis. Int. J. Pharm..

[27] Choudhary V., Shivakumar H. (2018). A review on curcumin: Wound healing properties and biomarkers of wound healing. Int. Res. J. Pharm..

[28] Barchitta M., Maugeri A., Favara G., Magnano San Lio R., Evola G., Agodi A., Basile G. (2019). Nutrition and wound healing: An overview focusing on the beneficial effects of curcumin. Int. J. Mol. Sci..

[29] Akbik D., Ghadiri M., Chrzanowski W., Rohanizadeh R. (2014). Curcumin as a wound healing agent. Life Sci..

[30] Mohanty C., Sahoo S.K. (2017). Curcumin and its topical formulations for wound healing applications. Drug Discov. Today.

[31] Verma S., Lan Y., Gokhale R., Burgess D.J. (2009). Quality by design approach to understand the process of nanosuspension preparation. Int. J. Pharm..

[32] Lee M.K. (2020). Liposomes for Enhanced Bioavailability of Water-Insoluble Drugs: In Vivo Evidence and Recent Approaches. Pharmaceutics.

[33] Shin G.H., Chung S.K., Kim J.T., Joung H.J., Park H.J. (2013). Preparation of chitosan-coated nanoliposomes for improving the mucoadhesive property of curcumin using the ethanol injection method. J. Agric. Food Chem..

[34] Rao S., Song Y., Peddie F., Evans A.M. (2011). Particle size reduction to the nanometer range: A promising approach to improve buccal absorption of poorly water-soluble drugs. Int. J. Nanomed..

[35] Kanicky J.R., Shah D.O. (2002). Effect of degree, type, and position of unsaturation on the pKa of long-chain fatty acids. J. Colloid. Interface Sci..

[36] Shaker S., Gardouh A.R., Ghorab M.M. (2017). Factors affecting liposomes particle size prepared by ethanol injection method. Res. Pharm. Sci..

[37] Wang D.K., Zhang C.X., Zhang W.T., Zhao C.H., Guan S.X. (2008). Characteristics and pharmacokinetics of docetaxel liposome containing Tween 80 or PEG-DSPE. J. Drug Deliv. Sci. Technol..

[38] Schober P., Boer C., Schwarte L.A. (2018). Correlation Coefficients: Appropriate Use and Interpretation. Anesth. Analg..

[39] Csicsák D., Szolláth R., Kádár S., Ambrus R., Bartos C., Balogh E., Antal I., Köteles I., Tőzsér P., Bárdos V. (2023). The effect of the particle size reduction on the biorelevant solubility and dissolution of poorly soluble drugs with different acid-base character. Pharmaceutics.

[40] Marwah M., Badhan R.K.S., Lowry D. (2022). Development of a novel polymer-based carrier for deformable liposomes for the controlled dermal delivery of naringenin. J. Liposome Res..

[41] Ameri M., Al-Mudhaffer M.F., Almyahi F., Fardell G.C., Marks M., Al-Ahmad A., Fahy A., Andersen T., Elkington D.C., Feron K. (2019). Role of stabilizing surfactants on capacitance, charge, and ion transport in organic nanoparticle-based electronic devices. ACS Appl. Mater. Interfaces.

[42] Muneer R., Hashmet M.R., Pourafshary P., Shakeel M. (2023). Unlocking the power of artificial intelligence: Accurate zeta potential prediction using machine learning. Nanomaterials.

[43] Masoumi H.R., Basri M., Samiun W.S., Izadiyan Z., Lim C.J. (2015). Enhancement of encapsulation efficiency of nanoemulsion-containing aripiprazole for the treatment of schizophrenia using mixture experimental design. Int. J. Nanomed..

[44] Pornpitchanarong C., Akkaramongkolporn P., Nattapulwat N., Opanasopit P., Patrojanasophon P. (2024). Development and Optimization of Andrographis paniculata Extract-Loaded Self-Microemulsifying Drug Delivery System Using Experimental Design Model. Pharmaceutics.

[45] Ahmad N., Khalid M.S., Al Ramadhan A.M., Alaradi M.Z., Al Hammad M.R., Ansari K., Alqurashi Y.D., Khan M.F., Albassam A.A., Ansari M.J. (2023). Preparation of melatonin novel-mucoadhesive nanoemulsion used in the treatment of depression. Polym. Bull..

[46] Nsairat H., Khater D., Sayed U., Odeh F., Al Bawab A., Alshaer W. (2022). Liposomes: Structure, composition, types, and clinical applications. Heliyon.

[47] Shariare M.H., Khan M.A., Al-Masum A., Khan J.H., Uddin J., Kazi M. (2022). Development of Stable Liposomal Drug Delivery System of Thymoquinone and Its In Vitro Anticancer Studies Using Breast Cancer and Cervical Cancer Cell Lines. Molecules.

[48] Battista S., Maggi M.A., Bellio P., Galantini L., D’Archivio A.A., Celenza G., Colaiezzi R., Giansanti L. (2020). Curcuminoids-loaded liposomes: Influence of lipid composition on their physicochemical properties and efficacy as delivery systems. Colloids Surf. A Physicochem. Eng. Asp..

[49] Pylypenko D., Gorbach T., Krasnopolsky Y. (2020). Study of antioxidant activity of liposomal forms of quercetin and curcumin in ischemic heart disease. BioTechnologia.

[50] Jakubczyk K., Druzga A., Katarzyna J., Skonieczna-Zydecka K. (2020). Antioxidant Potential of Curcumin-A Meta-Analysis of Randomized Clinical Trials. Antioxidants.

[51] Menon V.P., Sudheer A.R. (2007). Antioxidant and anti-inflammatory properties of curcumin. Adv. Exp. Med. Biol..

[52] Alven S., Nqoro X., Aderibigbe B.A. (2020). Polymer-Based Materials Loaded with Curcumin for Wound Healing Applications. Polymers.

[53] Comino-Sanz I.M., Lopez-Franco M.D., Castro B., Pancorbo-Hidalgo P.L. (2021). The Role of Antioxidants on Wound Healing: A Review of the Current Evidence. J. Clin. Med..

[54] Aye K.C., Rojanarata T., Ngawhirunpat T., Opanasopit P., Pornpitchanarong C., Patrojanasophon P. (2023). Development and optimization of curcumin-nanosuspensions with improved wound healing effect. J. Drug Deliv. Sci. Technol..

[55] Duangjit S., Opanasopit P., Rojanarata T., Ngawhirunpat T. (2013). Physicochemical stability of meloxicam loaded liposome formulation: Effect of cationic and anionic surfactant. Thai J. Pharm. Sci..

[56] Duangjit S., Obata Y., Sano H., Onuki Y., Opanasopit P., Ngawhirunpat T., Miyoshi T., Kato S., Takayama K. (2014). Comparative study of novel ultradeformable liposomes: Menthosomes, transfersomes and liposomes for enhancing skin permeation of meloxicam. Biol. Pharm. Bull..

[57] Hussein Y., El-Fakharany E.M., Kamoun E.A., Loutfy S.A., Amin R., Taha T.H., Salim S.A., Amer M. (2020). Electrospun PVA/hyaluronic acid/L-arginine nanofibers for wound healing applications: Nanofibers optimization and in vitro bioevaluation. Int. J. Biol. Macromol..

[58] Stauffer S.R., Peppast N.A. (1992). Poly(vinyl alcohol) hydrogels prepared by freezing-thawing cyclic processing. Polymer.

[59] Quintero S.M.M., Ponce F.R.V., Cremona M., Triques A.L.C., d’Almeida A.R., Braga A.M.B. (2010). Swelling and morphological properties of poly(vinyl alcohol) (PVA) and poly(acrylic acid) (PAA) hydrogels in solution with high salt concentration. Polymer.

[60] Waresindo W.X., Luthfianti H.R., Priyanto A., Hapidin D.A., Edikresnha D., Aimon A.H., Suciati T., Khairurrijal K. (2023). Freeze–thaw hydrogel fabrication method: Basic principles, synthesis parameters, properties, and biomedical applications. Mater. Res. Express.

[61] Lazic Z.R. (2006). Design of Experiments in Chemical Engineering: A Practical Guide.

[62] Pornpitchanarong C., Rojanarata T., Opanasopit P., Ngawhirunpat T., Patrojanasophon P. (2020). Synthesis of novel N-vinylpyrrolidone/acrylic acid nanoparticles as drug delivery carriers of cisplatin to cancer cells. Colloids Surf. B Biointerfaces.

[63] Dou X., Wang H., Yang F., Shen H., Wang X., Wu D. (2023). One-Step Soaking Strategy toward Anti-Swelling Hydrogels with a Stiff “Armor”. Adv. Sci..

[64] Wu F., Pang Y., Liu J. (2020). Swelling-strengthening hydrogels by embedding with deformable nanobarriers. Nat. Commun..

[65] Liu P., Chen G., Zhang J. (2022). A review of liposomes as a drug delivery system: Current status of approved products, regulatory environments, and future perspectives. Molecules.

[66] Pornpitchanarong C., Rojanarata T., Opanasopit P., Ngawhirunpat T., Bradley M., Patrojanasophon P. (2022). Maleimide-functionalized carboxymethyl cellulose: A novel mucoadhesive polymer for transmucosal drug delivery. Carbohydr. Polym..

[67] Zhang Y., Huo M., Zhou J., Zou A., Li W., Yao C., Xie S. (2010). DDSolver: An add-in program for modeling and comparison of drug dissolution profiles. AAPS J..

[68] Jayachandran P., Ilango S., Suseela V., Nirmaladevi R., Shaik M.R., Khan M., Khan M., Shaik B. (2023). Green synthesized silver nanoparticle-loaded liposome-based nanoarchitectonics for cancer management: In vitro drug release analysis. Biomedicines.

[69] Zhu W., Long J., Shi M. (2023). Release Kinetics Model Fitting of Drugs with Different Structures from Viscose Fabric. Materials.

[70] Masson-Meyers D.S., Andrade T.A.M., Caetano G.F., Guimaraes F.R., Leite M.N., Leite S.N., Frade M.A.C. (2020). Experimental models and methods for cutaneous wound healing assessment. Int. J. Exp. Pathol..

[71] Gopinath D., Ahmed M.R., Gomathi K., Chitra K., Sehgal P.K., Jayakumar R. (2004). Dermal wound healing processes with curcumin incorporated collagen films. Biomaterials.

[72] Hussain Y., Alam W., Ullah H., Dacrema M., Daglia M., Khan H., Arciola C.R. (2022). Antimicrobial Potential of Curcumin: Therapeutic Potential and Challenges to Clinical Applications. Antibiotics.

[73] Gounden V., Singh M. (2024). Hydrogels and Wound Healing: Current and Future Prospects. Gels.

[74] Khaleghi M., Haghi F., Gholami M., Hourfar H., Shahi F., Mir Mousavi Zekoloujeh A., Aliakbari F., Ahmadi E., Morshedi D. (2023). A fabricated hydrogel of hyaluronic acid/curcumin shows super-activity to heal the bacterial infected wound. AMB Express.

[75] Chopra H., Bibi S., Mohanta Y.K., Kumar Mohanta T., Kumar S., Singh I., Saad Khan M., Ranjan Rauta P., Alshammari A., Alharbi M. (2023). In Vitro and In Silico Characterization of Curcumin-Loaded Chitosan–PVA Hydrogels: Antimicrobial and Potential Wound Healing Activity. Gels.

[76] Le T.T., Nguyen T.K., Nguyen V.M., Dao T.C., Nguyen H.B., Dang C.T., Le T.B., Nguyen T.K., Nguyen P.T., Dang L.H. (2023). Development and Characterization of a Hydrogel Containing Curcumin-Loaded Nanoemulsion for Enhanced In Vitro Antibacteria and In Vivo Wound Healing. Molecules.

[77] Bhairy S., Hirlekar R., Dekate S. (2018). Preparation and Characterization of Oral Nanosuspension Loaded with Curcumin. Int. J. Pharm. Pharm. Sci..

[78] Eakwaropas P., Myat Y.Y., Ngawhirunpat T., Rojanarata T., Patrojanasophon P., Akkaramongkolporn P., Opanasopit P. (2019). Optimization of boesenbergia rotunda extract-loaded polyvinyl alcohol hydrogel wound dressing by box-behnken design. Key Eng. Mater..

[79] Pornpitchanarong C., Rojanarata T., Opanasopit P., Ngawhirunpat T., Patrojanasophon P. (2020). Clotrimazole nanosuspensions-loaded hyaluronic acid-catechol/polyvinyl alcohol mucoadhesive films for oral candidiasis treatment. J. Drug Deliv. Sci. Technol..

