# Supplementary material for: Computational Designed and Optimized Liposomal Curcumin-Embedded Bifunctional Cross-Linked Hydrogels for Wound Healing
Source: Gels. 2024 Sep 18;10(9):598. doi: 10.3390/gels10090598 (PMC11431055; doi:10.3390/gels10090598)
Supplement: Supplementary file 1 [file gels-10-00598-s001.zip › gels-3206452-supplementary.pdf]

†Electronic supplementary information

Computational designed and optimized liposomal curcumin-embedded bifunctional crosslinked hydrogels for wound healing

Chaiyakarn Pornpitchanarong[1], Khin Cho Aye[1], Kwanputtha Arunprasert[1,2], Nakarin Thammasalee[1], Bovornson Junjua[1], Varinthorn Chaovikrome[1], Praneet Opanasopit[1], Prasopchai Patrojanasophon[1,*]

[1]Pharmaceutical Development of Green Innovations Group (PDGIG), Faculty of Pharmacy, Silpakorn University, Nakhon Pathom, 73000, Thailand

[2]Health Intervention and Technology Assessment Program (HITAP), Ministry of Public Health, Nonthaburi, 11000, Thailand

General characterizations of the synthesized product

Nuclear magnetic resonance spectroscopy (NMR)

Molecular structure of the synthesized PNVP-ITA was examined using ^{1}H-NMR spectroscopy. The spectra were collected with NMR 300 MHz (AVANCE III HD, Bruker) spectrometer at 298 K. All chemical shifts were reported as δ in parts per million (ppm), using the chemical shift of D_2O (δ = 4.80 ppm) as reference.

Attenuated Total Reflectance-Fourier-transform infrared spectroscopy (ATR-FTIR)

The chemical structure of PNVP-ITA was confirmed by ATR-FTIR spectrophotometer (Nicolet iS5, Thermo Fisher Scientific, MA, USA). The spectrum with wavenumber ranged from 4000 to 500 cm^{-1} were collected with a resolution of 4 cm^{-1} and a total of 32 scans/run.

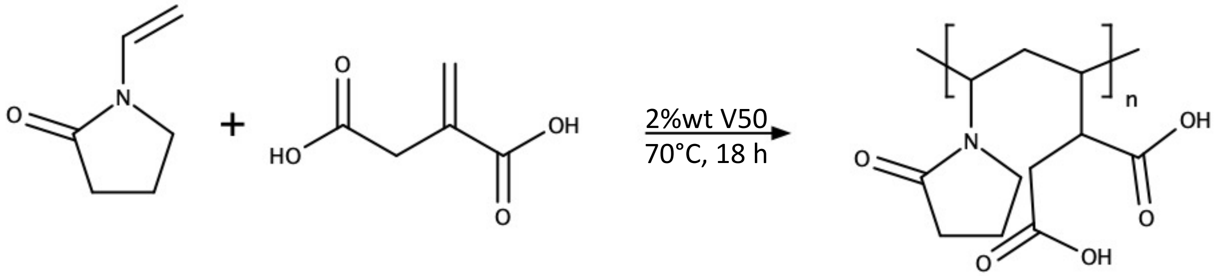

NVP ITA PNVP-ITA

Figure S1. Synthesis scheme of PNVP-ITA

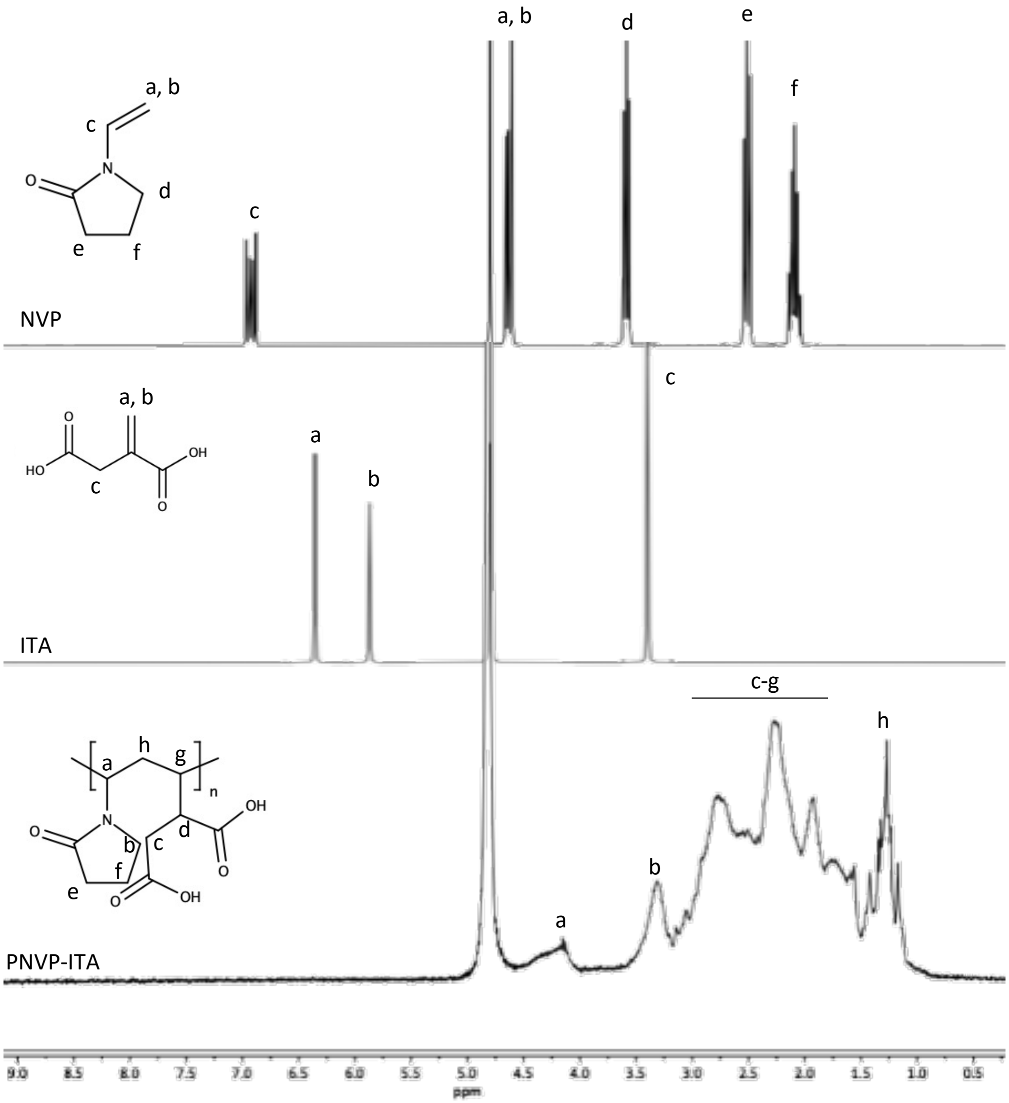

Figure S2. ^{1}H-NMR spectra of NVP, ITA, PNVP-ITA

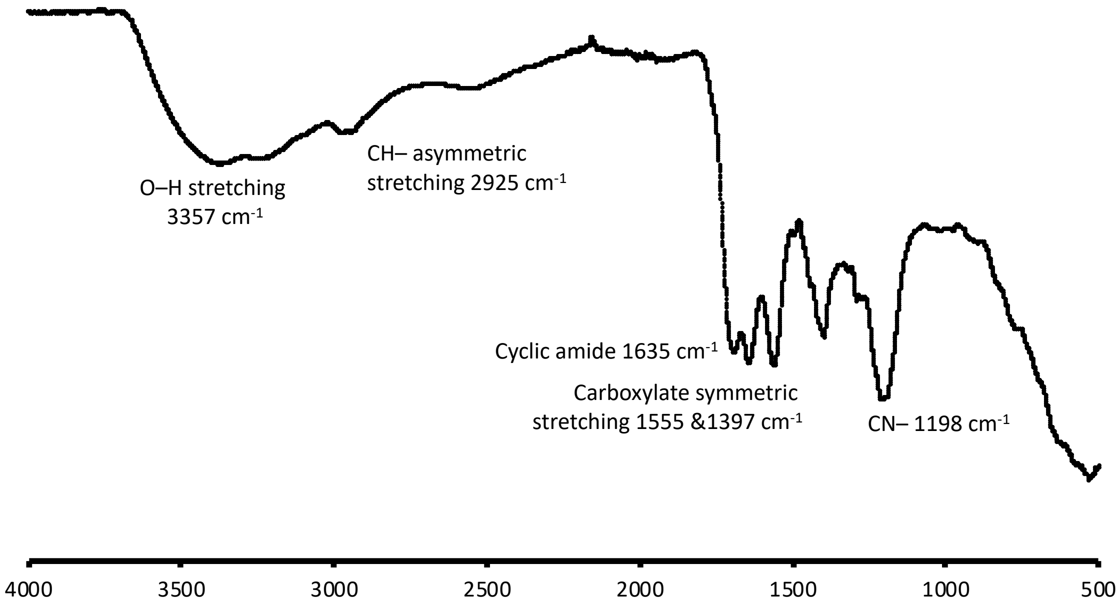

Figure S3. ATR-FTIR spectrum of PNVP-ITA

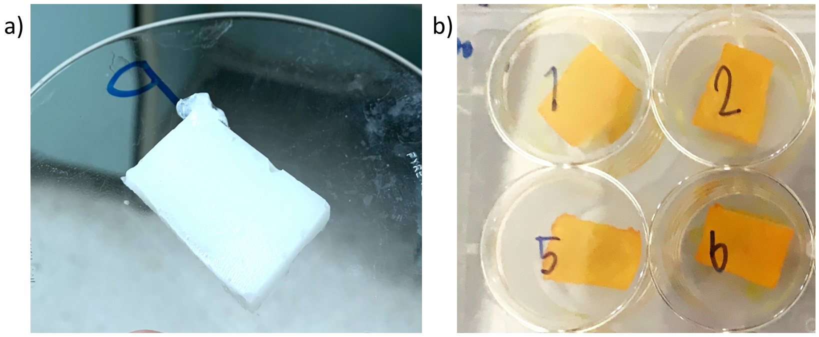

Figure S4 . The photograph of a) the blank HA/PVA/PNVP-ITA hydrogel and b) CUR-L@HA/PVA/PNVP-ITA hydrogel
